# Early Diagnosis and Cardiac Complications of Kawasaki Disease in a Resource-Limited Regional Hospital

**DOI:** 10.7759/cureus.82205

**Published:** 2025-04-13

**Authors:** Tawfiq Al Lawati, Hoor y Al Maharbi, Rokia A Al Zakwani, Riham K Al Nadhairi, Reem A Al Malki, Salah Al Awaidy

**Affiliations:** 1 Pediatrics, Al Rustaq Hospital, Al Rustaq, OMN; 2 Pediatrics, National University, Al Rustaq, OMN; 3 Epidemiology and Public Health, Oman Ministry of Health, Muscat, OMN

**Keywords:** a coronary artery anomaly, children, clinical and laboratory characteristics, clinical symptoms, early disease detection, intravenous immunoglobulin (ivig), kawasaki disease (kd), regional hospital, ” “resistance”, rural hospital

## Abstract

Introduction

Kawasaki disease (KD) is a type of vasculitis that mainly affects young children and is treated effectively with intravenous immunoglobulin (IVIG) when given promptly. Coronary artery aneurysm (CAA) is the most significant complication, occurring in 25% of untreated cases. However, this risk drops to 3% with IVIG treatment. This study aims to describe the clinical characteristics and cardiac complications of children diagnosed with KD at Al Rustaq Hospital (ARH).

Methods

A retrospective study was conducted on children under 13 years of age diagnosed with KD between January 1, 2010 and December 31, 2023. Data were retrieved from the hospital’s electronic records using the ICD-10 diagnostic code and cross-checked with pharmacy records of IVIG administration. Anthropometric, clinical, radiological, and biochemical data were collected and analyzed.

Results

Fifty children were identified, including 27 males (54.0%). The median age at presentation was 24 months (IQR: 14-43), and the median duration of fever was four days (IQR: 3-6). Diagnosis and initiation of IVIG treatment occurred at a median of two days (IQR: 1-3). Eight children (16.0%) were resistant to the first dose of IVIG. No significant differences were found between IVIG-resistant and IVIG-responsive groups in terms of age at diagnosis, fever duration, timing of IVIG administration, CRP, serum albumin, or alanine aminotransferase levels. The median CRP level significantly decreased from 143.3 mg/L before IVIG to 62.4 mg/L after treatment (p < 0.001). Six children (12.0%) had CAA on initial echocardiography, with only three (6.0%) showing persistent CAA on follow-up echocardiography eight weeks later. No significant associations were observed between CAA development and age at diagnosis, fever duration, CRP levels, albumin levels, or timing of IVIG administration.

Conclusions

KD is rarely encountered in regional hospital settings. Nonetheless, patients at ARH presented early, and diagnosis and treatment were initiated promptly despite the limitations of a secondary care facility. This early recognition and rapid management likely contributed to reducing long-term cardiac complications in children with KD.

## Introduction

Kawasaki disease (KD) is an idiopathic inflammatory condition that primarily affects small- and medium-sized blood vessels in infants under the age of five [1]. It is the leading cause of acquired coronary heart disease in adolescents, affecting up to 30% of untreated cases [2]. Diagnosis is based on the presence of fever along with at least four of the following five features: cervical lymphadenopathy, non-purulent conjunctivitis, oral mucosal changes, changes in the extremities or perineal skin, and a polymorphous rash [3].

Coronary artery aneurysm (CAA) is the most serious complication of KD, occurring in approximately 15-25% of untreated cases globally [4]. Early diagnosis and prompt administration of intravenous immunoglobulin (IVIG) significantly reduce this risk - from around 25% to 3-5% - representing a 75% decrease in incidence [5,6]. However, despite early treatment, about 20% of KD patients do not respond to the initial IVIG dose [4]. In such cases, a second dose is typically required, which prolongs hospital stays and increases the risk of CAA development [7].

Regional hospitals are often the first point of care for KD patients, particularly those from rural areas. Early recognition and timely management are essential to prevent long-term complications. However, limited resources in these settings can hinder early diagnosis. A multicenter study involving 13 Arab countries and 30 pediatric rheumatologists and cardiologists found that 60% of participants rated healthcare services in rural areas as either poor or fair [8].

Al Rustaq Hospital (ARH), located in a mountainous area roughly 120 kilometers from Muscat, serves as a general hospital providing secondary-level care to 518,026 individuals - 38.4% of whom are children under the age of 15 [9]. The hospital lacks both a pediatric cardiologist and a pediatric rheumatologist. All children requiring echocardiography must be referred to tertiary centers in Muscat.

In a seven-year study beginning in 2003, Bhatnagar et al. followed 39 children diagnosed with KD at a leading tertiary hospital in Oman. The mean fever duration was around nine days; 8% were resistant to the initial IVIG dose, and 25% developed CAA within six to eight weeks of illness [10]. Similarly, Al Mwaiti et al. reviewed cases from the only two tertiary care hospitals in Oman and reported a CAA prevalence of 26% on initial echocardiogram and 18% at follow-up after six to eight weeks [11]. Neither study offered an explanation for the high rate of cardiac complications observed.

Currently, no data compare the rate of KD recognition and CAA development between regional secondary-level hospitals and better-equipped tertiary care centers.

We hypothesize that delayed presentation and referral of KD patients in remote areas may contribute to the prolonged fever duration and elevated CAA rates reported in tertiary settings. This study aims to describe the timing of KD detection at ARH and the prevalence of CAA at six to eight weeks of follow-up.

The objectives of the study are to describe the clinical characteristics of children with KD, determine the timing of IVIG administration, assess the rate of resistance to the first IVIG dose, and evaluate echocardiographic findings at both initial and follow-up visits in a regional hospital with limited secondary care capacity.

## Materials and methods

Study design and participants

This retrospective cohort study was conducted at ARH from January 1, 2010 to December 31, 2023. All individuals under the age of 13 who were diagnosed with KD during this period were included. Children older than 13 years were excluded, as they are considered adults in the Omani medical system and are managed by adult physicians. KD diagnosis followed worldwide standards, and treatment was based on standard therapy with IVIG and acetylsalicylic acid [3]. Both typical and atypical KD cases were included. Upon diagnosis, all children were referred to a tertiary care facility for echocardiography, and they also received follow-up echocardiography six to eight weeks later at the same institution. IVIG resistance was defined as the persistence or recurrence of fever 48 hours after completing IVIG treatment [12].

Data collection

Data were obtained from the Ministry of Health electronic database in Oman, which uses the ICD-10 classification system. The medical records of all children diagnosed with KD were reviewed in detail. Clinical data were discussed with the primary investigators, and the accuracy of the collected information was cross-checked. Pharmacy records were also examined to confirm which children received IVIG and were matched against the list of KD cases identified via the ICD-10 diagnosis.

Demographic information, including age, sex, and residence, was collected. Clinical features such as fever, eye examination findings, lymphadenopathy, rash, and mucosal membrane changes were documented. Additionally, complications affecting the central nervous system, gastrointestinal system, and urinary system were recorded. Laboratory results - including CRP, hemoglobin (Hb), WBC count, platelet count, liver and renal function tests, chest X-rays, abdominal ultrasounds (if performed), and echocardiographic findings - were gathered for both the initial assessment and the six- to eight-week follow-up.

All children under the age of 13 diagnosed with KD or mucocutaneous lymph node syndrome were included in the study.

Statistical analysis

Statistical analysis was conducted using IBM SPSS Statistics for Windows, Version 20.0 (Released 2011; IBM Corp., Armonk, NY, USA). Continuous numeric data were tested for normality before statistical analysis. Results were reported as mean with SD or median with IQR. The Student’s t-test was used to evaluate relationships for continuous parametric data, and the chi-square test was used for categorical data. The nonparametric Mann-Whitney U test was employed to assess differences in clinical parameters between the resistant and non-resistant groups to the first dose of IVIG. Binary logistic regression was used to identify risk factors for CAA, with results reported as ORs, 95% CIs, and p-values.

Ethical approval

Ethical approval was obtained from the Health Studies and Research Approval Committee, Ministry of Health, Sultanate of Oman (approval number MoH/CSR/24/27978). Patient consent was not required, as the study was anonymous and retrospective. The research adhered to the ethical standards set by the institution and the country and complied with the Helsinki Declaration.

## Results

Over the 13-year study period, 50 children were diagnosed with KD. Of these, 27 (54%) were male and 23 (46%) were female. The median age at presentation was 24 months (IQR = 14-43). Fever was the most common presenting symptom, reported in 49 children (98%), with a median duration of four days (IQR = 3-6). Conjunctivitis was observed in 43 children (86%), and rash was reported in 41 (82%). Additionally, 21 children (42%) presented with cough and rhinorrhea, and 11 (22%) experienced diarrhea. Irritability was noted in 29 children (58%) as a prominent clinical feature. Table 1 summarizes the frequencies of all recorded clinical symptoms.

**Table 1:** Prevalence of clinical signs and symptoms in patients with KD BCG, Bacillus Calmette-Guérin; KD, Kawasaki disease

Category	Number of patients (n = 50)	Percentage
Duration of fever (days)		
2-3	14	28
4-5	9	18
5-6	14	28
7-8	6	12
>7	4	8
Missing	3	6
Skin and mucosal findings		
Conjunctivitis	43	86
Extremities changes	17	34
Oral mucosal changes	40	80
BCG scar flare	12	24
Peri anal excoriation	11	22
Cervical lymphadenopathy		
No lymphadenopathy	1	2
Bilateral	15	30
Unilateral	16	32
Missing	18	36
Nervous system signs and symptoms		
No signs or symptoms	15	30
Irritability	29	58
Headache	2	4
Seizures	2	4
Missing	2	4
Urinary system signs and symptoms		
No signs or symptoms	39	78
Pyuria	8	16
Missing	3	6
Respiratory system signs and symptoms		
No signs or symptoms	27	54
Cough	14	28
Rhinorrhea	7	14
Missing	2	4
GI signs and symptoms		
No GI symptoms	24	48
Abdominal pain	1	2
Diarrhea	11	22
Vomiting	10	20
Missing	4	8

Blood investigations showed a mean Hb level of 10.3 g/dL (SD ± 1.0) and a mean CRP level of 143.3 mg/L (SD ± 107.6) prior to IVIG treatment. Following the first dose of IVIG, the mean CRP level significantly decreased to 62.4 mg/L (SD ± 57.5), with a p-value of <0.001. Elevated alanine aminotransferase (ALT) levels were observed in 20 children (40%). Table 2 provides a detailed summary of the relevant blood investigations.

**Table 2:** Relevant laboratory results of patients with KD ALT, alanine aminotransferase; AST, aspartate aminotransferase; ESR, erythrocyte sedimentation rate; Hb, hemoglobin; IVIG, intravenous immunoglobulin; KD, Kawasaki disease

Laboratory test	Reference range	Mean ± SD or median (IQR)
Mean Hb	11.5-15.5 g/dL	10.1 ± 1.0
Median WBC count	4.5-14.5 × 10⁹/L	12.96 (9.46-14.25)
Mean platelets	150-400 × 10⁹/L	335.6 ± 110.0
Mean CRP before IVIG	<10.0 mg/L	143.3 ± 107.6
Mean CRP after IVIG	<10.0 mg/L	62.4 ± 57.5
Mean ESR before IVIG	≤10 mm/hour	64.1 ± 32.2
Mean albumin	35-55 g/L	29.7 ± 6.4
Mean sodium	135-145 mmol/L	131.9 ± 2.2
Median AST	8-33 IU/L	42.5 (34.95-59.72)
Median ALT	4-36 IU/L	36.87 (13.0-91.8)

The median time to the first dose of IVIG administration was 2.0 days (IQR = 1-3) from admission. Eight children (16%) were resistant to the initial IVIG treatment and required a second dose. No child developed resistance to the second dose of IVIG, and none required third-line biological treatment. Of the eight children with IVIG resistance, two developed CAA, while six had normal echocardiography findings at the six- to eight-week follow-up (p = 0.063). There were no significant differences in age at diagnosis, duration of fever, timing of IVIG administration, CRP, serum albumin, or ALT levels between the IVIG-responsive and IVIG-resistant groups (Table 3).

**Table 3:** Difference in relevant clinical parameters between first-dose IVIG-resistant and IVIG-responsive groups IVIG, intravenous immunoglobulin

Clinical variable	Resistant group (n = 8)	95% CI	Responsive group (n = 42)	95% CI	p-Value
Age of diagnosis (months)	25.0 ± 21.62	6.9-43.1	34.0 ± 30.9	24.2-43.7	0.481
Duration of fever (days)	3.5 ± 2.4	1.5-5.6	4.8 ± 2.5	4.0-5.6	0.16
CRP before IVIG (mg/dl)	203.4 ± 118.5	104.3-302.4	143.6 ± 94.9	111.9-175.2	0.18
Albumin (g/l)	28.3 ± 5.8	22.2-34.3	30.1 ± 6.9	27.8-32.3	0.386
Timing of IVIG administration (days)	2.25 ± 1.5	1.0-3.5	2.4 ± 1.9	1.8-2.9	0.989

Coronary artery abnormalities were identified in six children (12%) during the initial echocardiography, which was conducted at a median time of 10 days (IQR = 4-28) after admission. Myocarditis was not detected in any of the patients. Follow-up echocardiography, performed at a median time of 40.5 days (IQR = 25-74), showed persistent CAA in three children (6%). Table 4 presents the association between relevant clinical parameters and the development of CAA six to eight weeks after initial presentation.

**Table 4:** Association between relevant clinical variables and the development of CAA eight weeks after presentation CAA, coronary artery aneurysm; IVIG, intravenous immunoglobulin

Clinical variable	Without CAA (N = 47)	95% CI	With CAA (N = 3)	95% CI	OR	p-Value
Mean age of diagnosis (± SD)	36.3 (±33.9)	24.3-49.0	13.3 (±10.4)	12.5-39.2	1	0.735
Mean duration of fever (± SD)	5.3 (±2.9)	4.1-6.4	2.7 (±1.5)	1.5-6.1	1.2	0.633
Mean CRP value before IVIG (± SD)	131.1 (±89.0)	96.9-166.0	168.2 (±38.9)	71.4-265.1	1	0.999
Mean albumin level (± SD)	29.5 (±6.7)	26.8-32.3	32.2 (±39.0)	23.1-41.2	0.9	0.826
Mean time of first IVIG administration (days)	2.0 (±1.6)	1.4-2.6	2.7 (±1.2)	0.20-5.5	0.9	0.9

## Discussion

The current study emphasizes the relatively early presentation, rapid detection, and swift administration of IVIG in a community hospital setting. It also highlights the high incidence of atypical symptoms, such as cough, runny nose (40%), and diarrhea (20%), which may initially mislead clinicians into diagnosing a viral illness. Furthermore, the study reveals that the rate of resistance to the first dose of IVIG (16%) and the occurrence of long-term cardiac complications (6%) are comparable to those observed in international studies [13].

These findings challenge the initial hypothesis that children with KD in rural and regional hospitals present later, leading to delayed diagnoses.

The short fever duration (four days) observed in our study contrasts with Bhatnagar et al.’s study [10], where the mean fever duration was nine days. Similarly, in our cohort, the mean fever duration was shorter than the eight-day duration (ranging from four to 21 days) reported by King Fahad University in Saudi Arabia [14]. Additionally, the fever duration in our cohort was shorter than the 7.2 days (IQR = 5-25.9) reported in a recent study of 40 children in Newcastle, Australia [15]. This short duration of fever reflects early patient presentation to health services, countering the assumption that patients in rural areas present late.

The administration of IVIG within 48 hours of presentation is relatively quick, particularly since 40% of patients exhibited atypical features like a runny nose and diarrhea. This rapid identification and treatment initiation likely reflects a high level of awareness of KD among the pediatricians involved. However, comparisons to other studies regarding the timing of IVIG administration are difficult due to the lack of similar data. We also recognize that some patients may have initially been misdiagnosed with a viral illness when they actually had KD. Misdiagnosis in KD has been reported as high as 25%, as seen in Latin America [16].

An interesting finding was that 12 patients (24%) experienced a flare of the Bacillus Calmette-Guérin (BCG) scar, which is now considered a diagnostic criterion for skin changes in the new Japanese guidelines [17]. The reported prevalence of BCG scar flare ranges from 30% to 50%, which is slightly higher than our study’s findings [18].

Another noteworthy observation was the long median time to the initial echocardiogram, which occurred at 10 days (ranging from four to 28 days). This delay in obtaining echocardiograms for KD patients is likely due to the lack of an in-house cardiologist and reliance on external tertiary care centers for specialized imaging.

Our study also demonstrated a significant reduction in CRP levels after the administration of IVIG. Although CRP is not a diagnostic criterion for KD, the decrease in CRP following IVIG administration is a recognized feature that supports the diagnosis of KD [17].

Regarding resistance to the first dose of IVIG, our study reported a resistance rate of 16%, which is double that found in Bhatnagar et al.’s study (8%) [10]. Al Mwaiti et al. did not report the resistance rate in their study from two tertiary centers [11]. Our 16% resistance rate aligns with the 20% rate reported internationally [4]. However, further studies are needed to understand why our rate is higher than that in Bhatnagar et al.’s study.

As for cardiac complications, the initial echocardiogram in our study showed a 12% incidence of coronary artery abnormalities, which is consistent with Bhatnagar et al.’s findings [10]. However, unlike Bhatnagar et al.’s study, where 13% of patients had non-CAA cardiac abnormalities (including myocarditis and valvular lesions), we did not observe any other cardiac anomalies. Al Mwaiti et al. reported a CAA prevalence of 26% in the initial echocardiogram, which dropped to 18% on follow-up at six to eight weeks in two tertiary care centers in Oman [11]. The difference in long-term CAA rates may reflect a selection bias in tertiary care centers, where only more complicated cases are followed, possibly due to late referrals. Our CAA rates are consistent with international rates of 3-5% [3], but lower than those reported from Pakistan (32%) [13] and Los Angeles (20%) [5]. On the other hand, our rates are higher than those reported from Japan (9%) [18]. The differences in prevalence may be due to genetic and environmental factors, which influence CAA development in KD [19].

Resistance to the first dose of IVIG was not significantly associated with the duration of fever, CRP levels, albumin levels, or the timing of IVIG administration, which differs from other studies [20]. In contrast, some studies [21] found an association between the duration of fever, high platelet levels, low albumin, and male sex with the later development of CAA, but we did not observe this in our cohort. It is unclear why our study differed from others that showed such associations. A small sample size and potential genetic variations may contribute to these discrepancies, as seen in some studies [22].

The limitations of our study include missing data, such as the prevalence of cervical lymphadenopathy during physical examination and erythrocyte sedimentation rate values. Missing data is an inherent issue in retrospective studies, but it did not significantly affect our findings, as documenting physical signs was not the main aim. The relatively small patient population was another limitation; however, it reflects the total number of identified cases, not a sample. Nevertheless, the sample size remains larger than those of studies from regions like Pakistan and Los Angeles [13,5].

## Conclusions

KD is rarely encountered in regional hospital settings. Despite the limitations of secondary care environments, patients in our study presented early to healthcare facilities. The identification and initiation of treatment occurred relatively quickly. Early presentation, along with a high index of suspicion and effective, rapid management, likely contributed to the mitigation of long-term cardiac complications in this cohort.
